# Enhanced Injection Molding Simulation of Advanced Injection Molds

**DOI:** 10.3390/polym9020077

**Published:** 2017-02-22

**Authors:** Béla Zink, Ferenc Szabó, István Hatos, András Suplicz, Norbert Krisztián Kovács, Hajnalka Hargitai, Tamás Tábi, József Gábor Kovács

**Affiliations:** 1 Department of Polymer Engineering, Faculty of Mechanical Engineering, Budapest University of Technology and Economics, Műegyetem rkp. 3., H-1111 Budapest, Hungary; zink@pt.bme.hu (B.Z.); szabof@pt.bme.hu (F.Sz.); suplicz@pt.bme.hu (A.S.); kovacsn@pt.bme.hu (N.K.K.); tabi@pt.bme.hu (T.T.); 2 Department of Materials Science and Technology, Széchenyi István University, Egyetem tér 1, H-9026 Győr, Hungary; hatos@sze.hu (I.H.); hargitai@sze.hu (H.H.); 3 MTA–BME Research Group for Composite Science and Technology, Műegyetem rkp. 3., H-1111 Budapest, Hungary

**Keywords:** injection molding, direct metal laser sintering, injection molding simulation, conformal cooling, high conductivity copper alloy

## Abstract

The most time-consuming phase of the injection molding cycle is cooling. Cooling efficiency can be enhanced with the application of conformal cooling systems or high thermal conductivity copper molds. The conformal cooling channels are placed along the geometry of the injection-molded product, and thus they can extract more heat and heat removal is more uniform than in the case of conventional cooling systems. In the case of copper mold inserts, cooling channels are made by drilling and heat removal is facilitated by the high thermal conductivity coefficient of copper, which is several times that of steel. Designing optimal cooling systems is a complex process; a proper design requires injection molding simulations, but the accuracy of calculations depends on how precise the input parameters and boundary conditions are. In this study, three cooling circuit designs and three mold materials (Ampcoloy 940, 1.2311 (P20) steel, and MS1 steel) were used and compared using numerical methods. The effect of different mold designs and materials on cooling efficiency were examined using calculated and measured results. The simulation model was adjusted to the measurement results by considering the joint gap between the mold inserts.

## 1. Introduction

Injection molding has seen rapid progress in the past decades, and it is now one of the most important polymer processing technologies. The most significant phase of the injection molding cycle is cooling, which—in the case of large-volume products—high processing temperature or complicated geometry can amount to more than half of the entire cycle. With such products, a reduction in cooling time considerably improves productivity. One of the best ways to achieve this is to use mold inserts with conformal cooling. As opposed to a conventional cooling system, this system follows the geometry of the product; therefore, it can extract more heat and heat removal is also more uniform. This results in a reduction of cycle time and an improvement of product quality [1].

Direct metal laser sintering (DMLS) is similar to selective laser sintering (SLS), but its raw material is the powdered metal used to create the tool itself. DMLS technology is a direct additive manufacturing technology in which the surface of the metal powder is melted with a laser beam. DMLS is a continuously developing technology, and much research focuses on developing the technology [2,3,4,5] and materials [6,7,8]. 

Injection molding simulations are used to make proper cooling layouts, and it is even more important to use computer calculations for conformal cooling systems. The accuracy of the results is greatly influenced by the precision of modeling, boundary conditions, and the calculation algorithm. More and more articles deal with the development of the simulation results; Zhang et al. [9] developed a novel boundary element method (BEM)-based cooling simulation method for steady-state cooling. The analytical solution of the part temperature was introduced into the BEM, so the random access memory (RAM) size required for the calculations was reduced by 93% on average and calculation time was shortened by one order of magnitude. Liu and Gehde [10] analyzed the influence of the heat transfer coefficient (HTC) between the polymer and cavity wall on cooling and crystallinity. They found that melt temperature and surface roughness have important roles on the determination of the HTC. A difference between the frozen percentages calculated by the observed and preset HTC values were reported, and the HTC also influenced relative crystallinity.

The advantages of conformal cooling have been studied for some time now, and several studies have been published on the automatic generation of conformal cooling circuits. Michaeli and Schönfeld [1] stated that the advantage of conformal cooling compared to conventional cooling in the case of cylindrical mold inserts is less pronounced than in the case of angular inserts, where heat accumulates in the corners. Taylor et al. [11] stated that the use of conformal cooling can result in an increase in productivity by nearly 25% and in a reduction of energy use by 11%, but mold inserts manufactured with SLS are less durable than inserts manufactured with conventional technologies. Meckley and Edwards [12], Coremans et al. [13], and Ränner et al. [14] also investigated the differences between conventional and conformal cooling systems. They reported that conformal cooling systems can shorten the injection molding cycle significantly. Yu et al. [15] examined conformal cooling circuits generated automatically, with an algorithm. They concluded that even though such a cooling circuit contains many branches and therefore the coolant slows down, it can still extract more heat from the part than conventional layouts. However, they did not verify their calculations with measurements and used simple beam elements in their simulation; therefore, they could not take into account hydrodynamic processes in the cooling circuit. Studies so far have not focused on a comprehensive comparison of cooling systems and materials of different thermal conductivity. 

These novel molding methods are important in conventional injection molding technology, but it is even more important to achieve good parts using self-reinforced materials [16] or poly(lactic acid)-based materials [17].

In this paper, mold inserts of special shapes and materials are examined with injection molding simulation. Three types of cooling circuits were investigated: a cooling circuit made with conventional technology (most widely used in the industry), a cooling circuit assembled from several parts but also made with conventional technology (milling and drilling), and a conformal cooling system manufactured with DMLS technology. The effect of mold material on cooling was also investigated: conventional P20 steel, a high thermal conductivity copper alloy, and MaragingSteel MS1 were compared with regard to the spatial and surface thermal distribution of the mold inserts and the expected warpage of the product. Our aim was to compare the cooling efficiency of mold inserts of different materials and different cooling circuits using a numerical method in the case of a product which is of small volume and simple geometry, but still difficult to cool with a conventional cooling system.

## 2. Heat Transfer Equations

Right after the mold and injection unit is closed, the melt is injected into the tempered cavity at high speed until the cavity is fully filled. The melt starts to transmit energy to the mold right after it touches its wall. In the upcoming packing phase, some more melt is pushed into the cavity, so more heat is transmitted to the mold. Heat removal is highest in the injection phase because cavity pressure and the temperature difference between the melt near the wall and the cavity wall are at their highest. The pressure drops during the cooling stage until it reaches atmospheric pressure. Because of volumetric shrinkage, an air gap is formed between the cavity wall and the injection-molded part, so heat transfer between the injected part and the mold drops significantly. The melt flow and temperature distribution can be calculated with Navier–Stokes’ momentum (1), continuity (2), and energy (3) conservation equations [10,18]:

(1) $$\varrho \cdot \left(\frac{\partial v}{\partial t}+v\cdot \nabla v\right)=-\nabla p+\nabla \left(\eta \cdot \left(\nabla v+{\left(\nabla v\right)}^{T}\right)\right)-\frac{2}{3}\eta \cdot \nabla \left(\nabla v\right)+\varrho \cdot g,$$

(2) $$\frac{\partial \varrho }{\partial t}+\nabla \left(\varrho v\right)=0,$$

(3) $$\varrho \cdot c_{p}\cdot \left(\frac{\partial T}{\partial t}+v\cdot \nabla T\right)=\nabla \left(\lambda \cdot \nabla T\right)+T\cdot \beta \cdot \left(\frac{\partial p}{\partial t}+v\cdot \nabla T\right),$$

where

(4) $$\beta =-\frac{1}{\varrho }\frac{\partial p}{\partial T}.$$

**Figure 1:**
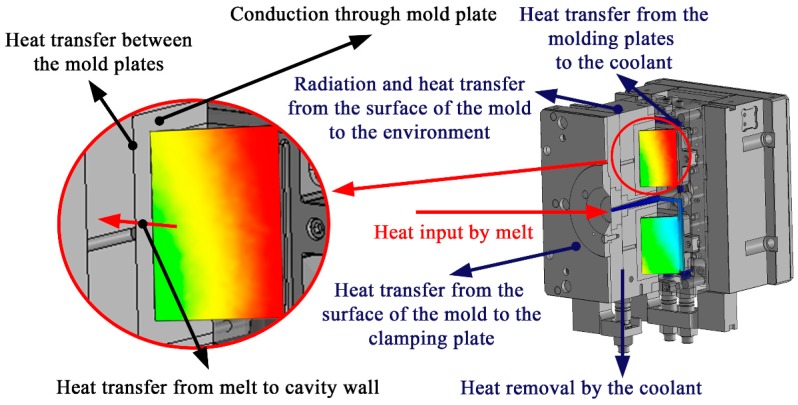
Heat transfer in the mold.

The heat from inside the cavity is removed by conduction, radiation, and heat transfer (Figure 1). This radiation (5), conduction (6), and convection (7) from the melt to the mold, clamping unit, and the atmosphere can be decomposed with the following equations [19]

(5) $$\dot{Q}=\int_{0}^{t_{cycle}}\sigma_{0}\cdot \varepsilon \cdot A\cdot \Delta T^{4}\cdot dt,$$

(6) $$\dot{Q}=\int_{0}^{t_{cycle}}\frac{\lambda }{\delta }\cdot A\cdot \Delta T\cdot dt,$$

(7) $$\dot{Q}=\int_{0}^{t_{cycle}}\alpha \cdot A\cdot \Delta T\cdot dt.$$

## 3. Experimental and Simulation

The purpose of the experiments was to investigate the effect of different cooling circuits and mold materials on the quality of the injection-molded part. A total of four mold inserts and cooling circuits were designed from three materials for the numerical procedures.

### 3.1. Simulation

The simulations were made with the Autodesk Simulation Moldflow Insight 2016 and CFD 2016 programs, using ABS (BASF, Terluran GP35, Ludwigshafen, Germany). Four-node tetrahedral elements were used in the entire model for the meshing. This makes it possible to consider the heat conduction in all directions and to calculate the temperature in all nodes. Global element size was set to 1.5 mm, but mesh size was changed to 1 mm in those areas where it was required by the complex geometry; for example, the gate and the cooling channels. The element number was nearly six million for each model. Cool finite element method (FEM) was used for the thermal analysis, which provides options to investigate the transient state of the mold. The conduction solver was used for the calculation of heat flux. Perfect clamping was assumed, and therefore mold block conductance was set to the default 30,000 W/m^2^·°C (Table 1). The effect of flow speed (4 and 8 L/min) and coolant temperature (40 and 20 °C) were also examined.

### 3.2. Mold Materials

Three mold materials were used in the experiments: high thermal conductivity Ampcoloy 940, MaragingSteel MS1, and 1.2311 (P20) steel, most often used for molds (Table 2).

### 3.3. Mold Design

**Figure 2:**
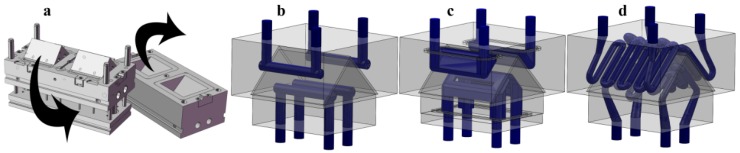
(**a**) The injection mold block used in the simulations, and the (**b**) Conventional, (**c**) Milled, and (**d**) Conformal cooling systems of the inserts.

Four different mold inserts were made in a two-cavity mold block for the cooling experiments (Figure 2a). Our reference was the setup most often used in industry, made from 1.2311 steel with a conventional cooling geometry (*P20* insert) (Figure 2b). The second mold insert was also made from conventional 1.2311 steel, but the mold insert was assembled from several parts and milled to better follow the surface (*Multi-part* insert) (Figure 2c). The third investigated mold insert was made from MS1 with a conformal cooling system by direct metal laser sintering (*DMLS* insert) (Figure 2d). The fourth mold insert also had a conventional cooling system, but was made from high thermal conductivity Ampcoloy 940 (*Ampcoloy* insert).

### 3.4. Temperature Measurent

The calculated surface temperature was checked by thermography measurements. The measurements were taken with a FLIR 325SC thermographic camera (Wilsonville, OR, USA). The emissivity of the insert ε = 0.3–0.6 and the average surface roughness *R*_a_ = 0.15–2 μm were low, so the surface of the inserts was painted with a high emissivity polymer film; therefore, reflectance was minimized and the emissivity of the surface was raised to ε_coating_ = 0.95. The viewing angle in all cases was near the optimum of α = 0°. The reflected apparent temperature was specified with a high reflectance aluminum foil (*T*_refl_ = 28.8 °C). The measurements were executed using the parameters of the simulation and were only started after 50 cycles to reach the equilibrium state. A minimum of three measurements were taken for every insert, and the results were averaged.

## 4. Results and Discussion

The effect of different mold layouts and materials on cooling efficiency and product quality were examined at identical simulation parameters.

### 4.1. Flow Analysis of Cooling Circuits

**Figure 3:**
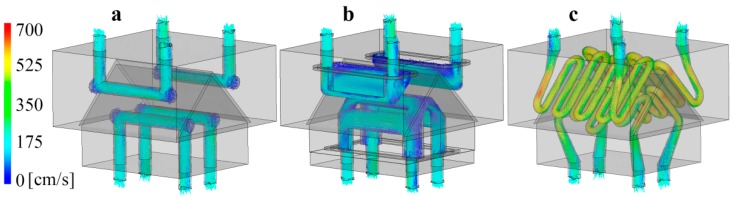
Flow velocity as a function of distance in the case of the (**a**) Conventional; (**b**) Milled multi-part; and (**c**) Conformal cooling system.

The flow velocity of the coolant (Figure 3) is a very important factor in determining cooling efficiency. The simulation shows significantly different flow velocity values for the different mold inserts. In the conventional mold insert, the coolant stagnates at the corners. This reduces heat transfer and increases the temperature of the stagnant coolant, therefore decreasing cooling efficiency (Figure 3a). In the *Multi-part* mold inserts, flow speed decreases considerably in several places, thus impairing cooling efficiency (Figure 3b). In the conformal cooling system, a reduction of diameter results in a noticeable increase in flow speed, which doubles the Reynolds number and the heat transfer coefficient (Figure 3c). The layout of conformal cooling circuits ensures that the flow of the coolant is uniform in critical parts of the insert; more coolant flows in these directions, and so the upper edge of the movable mold insert receives more coolant. Another difference between the cooling channels is surface finishing; the laser-sintered *DMLS* insert has notably higher roughness, which influences the turbulence of the flow. In addition, the cooling channels manufactured by DMLS technology corroded faster.

### 4.2. Examination of the Cross-Sectional Temperature of Mold Inserts

**Figure 4:**
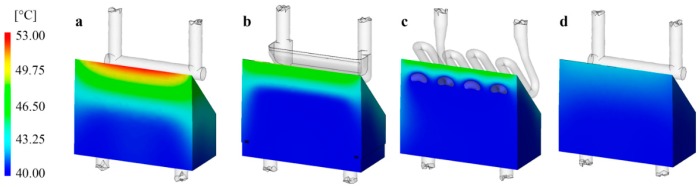
Averaged temperature distribution in the central cross-section of (**a**) *P20*, (**b**) *Multi-part*, (**c**) *DMLS*, and (**d**) *Ampcoloy* mold inserts.

Figure 4 shows the averaged temperature distributions of the center of mold inserts on the moving half. The maximum temperature of the conventional layout (used for reference) exceeds 52 °C at the edge of the insert furthest from the cooling circuits (Figure 4a). The maximum temperature of the mold insert with the milled multi-part cooling circuit is several degrees lower, and its temperature distribution is also more uniform (Figure 4b). In this case, similarly to the conventional mold insert, more heat accumulated at the edge of the insert, as the cooling system cannot transfer enough heat from this spot.

In the case of conformal cooling, the *DMLS* mold insert had a maximum temperature of 46 °C immediately next to the surface, the temperature under it was nearly 43 °C, but the rest of the cross-section had the same temperature as the coolant (Figure 4c). Compared to the mold insert of conventional layout, it can be seen that the temperature of the mold insert with conformal cooling also increased at the edge of the insert, but to a far lesser extent and in a far smaller volume since the cooling circuits are also located nearer the edge of the mold insert.

The last mold insert examined was the *Ampcoloy* insert (Figure 4d), which produced the most uniform temperature distribution of all the samples. The maximum temperature is the lowest in this case, nearly 10 °C lower than that of the conventional mold insert. Based on the results, it can be stated that the *Ampcoloy* mold insert has a better cooling efficiency than conformal cooling systems in the case of the examined mold, even with a conventional cooling circuit. However, this may not be true of more complex cooling circuit geometries, as the geometry of the part can be better followed with the DMLS technology, and its material is also more resistant to the mechanical stresses during injection molding.

Changing the temperature of the coolant did not result in significant changes in any of the layouts, but the temperatures shifted by 20 °C. The change in the flow speed of the coolant decreased temperature values by a few tenths of a degree in the case of each sample.

### 4.3. Evaluation of Surface Temperature

**Figure 5:**
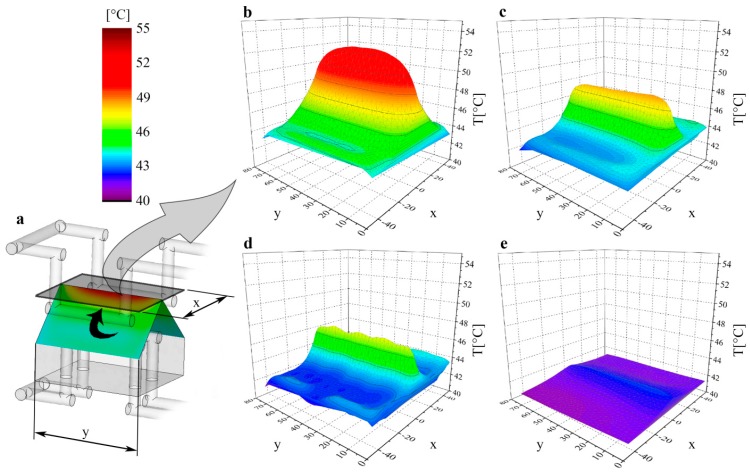
(**a**) Temperature distribution transformed into one plane in the case of the (**b**) *P20*, (**c**) *Multi-part*, (**d**) *DMLS*, and (**e**) *Ampcoloy* mold inserts.

The surface temperatures of the various mold inserts were also evaluated in our investigation. The coordinates of the surface of the mold inserts were transformed into one plane (Figure 5a), and the results displayed in a 3D graph. Thus, surface temperatures can be seen as a function of the *x*, *y* location coordinates.

Since the temperature axes are scaled identically, the results are easily comparable and show the efficiency of the various cooling systems well. When the *Multi-part* mold insert is compared to the *P20* insert, it can be seen (Figure 5b,c) that the maximum temperature is lower and the temperature is a few degrees higher only at the upper edge of the core, as the cooling circuits cannot transfer the accumulated heat from this part. The *DMLS* insert has a similar graph (Figure 5d), but there the average temperature and the maximum temperature are lower.

When the different mold insert materials and geometries are compared, it can be stated that the least efficient cooling circuit was the combination of tool steel and conventional cooling geometry, resulting in considerably higher average and maximum temperatures than the others. The best solution was the high thermal conductivity *Ampcoloy* insert with a conventional cooling geometry, where the maximum and average temperature hardly exceeded the temperature of the coolant. The two extremes showed a difference of 10 °C in maximum temperature and nearly 5 °C in average temperature (Table 3).

Surface temperature values changed similarly to cross-sectional temperatures as the temperature and flow speed were varied. The decrease in the temperature of the coolant did not bring about a significant change. An increase in the flow speed decreased the temperature slightly, and was more pronounced in parts near the cooling circuits; therefore, surface temperature differences increased. 

### 4.4. Measurement of Surface Temperature

**Figure 6:**
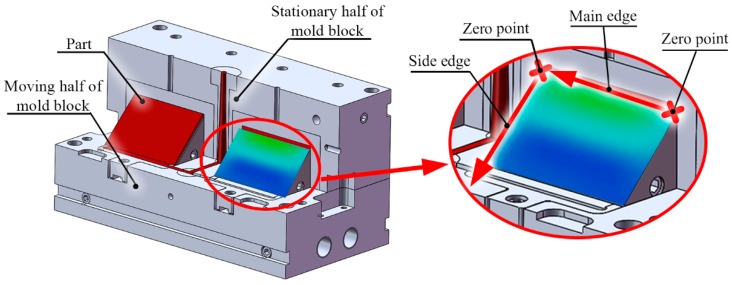
The notations used for the evaluation of the measurements, the side edge near the sprue and the main edge on the top of the moving insert.

The temperature results near the main and the side edge (which is nearer the sprue; Figure 6), were used for the evaluation. The temperature results were plotted as a function of the relative edge length; in the case of the side edge, the zero point is at the bottom of the edge. In the case of the main edge, the zero point is on the outer side of the insert measured from the sprue.

**Figure 7:**
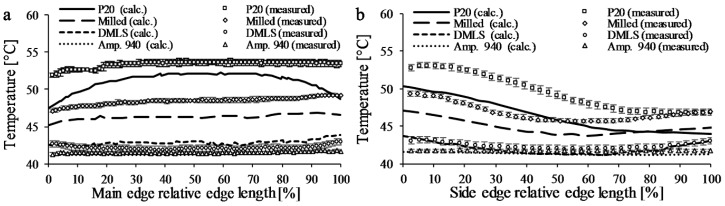
The measured and calculated surface temperature near (**a**) the main edge and (**b**) the side edge of the investigated inserts.

In all cases, the curves of the calculated and measured temperatures near the side edge of inserts are nearly similar; however, there is a 5 °C shift between the temperatures in the case of the *P20* and the *Multi-part* insert (Figure 7a,b). The temperature curves near the main edge are not similar for the *P20* and the *Multi-part* inserts; the calculated temperatures show a decrease at the beginning and the end of the edge, and the measured results have only a drop at the beginning of the curve. The reason for this difference can be measurement errors, but these errors were minimized, and for the *DMLS* and *Ampcoloy* inserts, the measured and the calculated results correlate well. The difference could be caused by the temperature fluctuation of the cooling water; the tolerance of the cooling unit was set to ±2 °C. Another possible reason may be the incorrect setting of the heat transfer coefficient between the moving and the stationary inserts and the inserts and the block, caused by the fitting of the inserts. Furthermore, the surface finishing of the mold influences the HTC value between the melt and the mold. Unfortunately, the inserts have a different surface roughness—the *P20* insert has the highest average surface roughness, *R*_a_ = 2 μm; this could also lead to a difference in temperature distribution. For the high cooling efficiency *Ampcoloy* insert and the *DMLS* insert, the heat transfer coefficient and coolant temperature play a smaller role, because the cooling efficiency of the molds is much higher. Coolant temperature and the joint gap between the inserts probably have the biggest impact on temperature distribution, because air has substantially lower heat conductivity and the heat transfer between the stationary air and the inserts is also low.

### 4.5. Investigating the Temperature Differences

**Figure 8:**
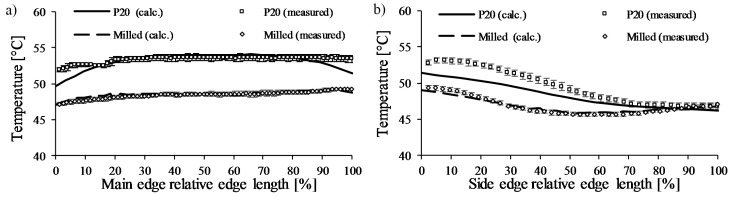
The temperature ramp of the *Multi-part* and *P20* insert in the case of coolant temperature 42 °C. Main edge (**a**); side edge (**b**).

Firstly, the influence of coolant temperature was investigated, and the temperature of the water was changed to 42 °C because the temperature was set to 40 ± 2 °C on the cooling unit. The results of the *Ampcoloy* insert with the modified coolant temperature show a good correlation between the measured and calculated mold surface temperatures on both the main and side edges (Figure 8). However, for the *P20* insert, there is a difference is between the calculated and the measured surfaces temperatures on the sides of the main edge and on the first 50% of the side edge, which can be caused by the incorrect setting of the heat transfer parameters on the sides.

**Figure 9:**
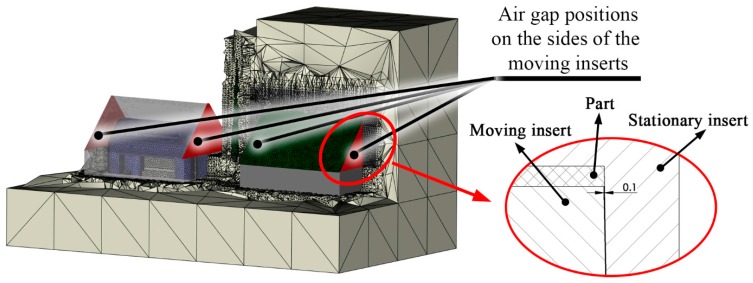
The positions of the air gaps in the simulation models.

A new model was set up with air gaps between the side faces of the core and cavity inserts (Figure 9). The fitting gap between the two inserts was measured by applying a high viscosity polymer liquid (dynamic viscosity around 600 Pas at room temperature), which provides an adequate contact surface between the inserts. The gap was found to be 0.1 ± 0.02 mm. The modeled gap has the thermal properties of air of 50 °C (Table 4). The thermal properties are temperature dependent, so we used the previously-run simulation to determine the temperature of the air.

**Figure 10:**
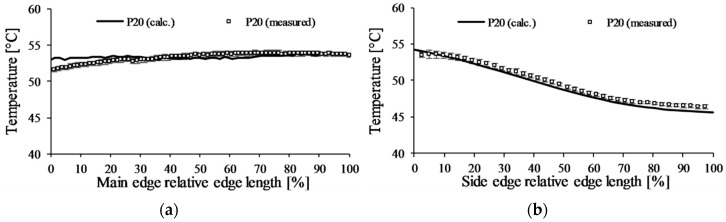
The temperature distribution of the *P20* insert with the corrected heat transfer along the (**a**) main and (**b**) side edge of the inserts.

The results show (Figure 10) that the temperatures retrieved from the model with the 0.1 mm air gap come closer to the measurement. The temperatures along the main edge are almost equal; the largest difference between the calculated and measured temperature results is around 1.5 °C. These temperature differences are in the tolerance field of the infrared camera, and can come from the surface roughness of the cooling channel and the cavity surface, which cannot be taken into account in simulations. These results show that in the case of an injection molding insert with lower cooling effectiveness (where the conduction of the mold material is significant), setting proper boundary conditions is essential.

## 5. Conclusions

We have shown that in many cases the cooling efficiency of complicated milled multi-part mold inserts falls considerably below expectations. The best way to improve cooling efficiency may be conformal cooling from the point of view of cooling circuit layout. We compared conventional and conformal cooling systems and materials of different thermal conductivity by using numerical methods and thermal measurements. We have concluded that even in the case of simple geometry parts, the use of conformal cooling systems instead of conventional cooling systems improves product quality, and reduces the cycle time and the heat load of the mold. Based on the results, it can be stated that copper-based highly alloyed mold materials can extract more heat even with a conventional cooling system in the case of simple and small injection molded parts than conformal cooling systems; therefore, these materials improve product quality more. The use of mold materials of good thermal conductivity is limited by their increased wear and deformation, but from a thermodynamic aspect, their application can be advantageous. The numerically calculated temperatures showed a great difference compared to the measured results in the case of the *Multi-part* and *P20* inserts, which have lower cooling efficiency. Heat removal is slower in these cases because of the lower thermal conductivity of the inserts; therefore, the boundary conditions have a greater influence on the calculations. The difference can be reduced by the proper setting of the boundary conditions, such as HTC, flow rate, temperature of the coolant, etc. HTC is affected by the joint air gaps, which form between the moving and stationary mold inserts. The results showed that the difference between the calculated and measured temperature results for the *P20* insert decreased significantly when the air gap was considered in the simulation model.

## Figures and Tables

**Table 1:** The simulation parameters used in the study.

Injection molding temperature (°C)	240
Ejection temperature (°C)	120
Ambient temperature (°C)	25
Mold surface temperature (°C)	40
Cooling time (s)	15
Initial mold temperature (°C)	40
Mold block conductance (W/m^2^·°C)	30,000
Number of heat flux time steps (-)	16
Transient mold temperature convergence tolerance (°C)	0.1
Maximum number of transient mold temperature cycles (-)	100

**Table 2:** The main characteristics of the materials used in the simulation.

Main characteristics	1.2311	Ampcoloy 940	MS1
Density (kg/m^3^)	7800	8710	8100
Tensile strength (MPa)	1020	544	1950
Yield point (MPa)	900	475	1900
Young’s modulus (GPa)	250	131	180
Thermal conductivity coefficient (W/mK)	29	208	20
Specific heat capacity (J/kgK)	460	380	450

**Table 3:** The maximum and average surface temperatures and the temperature differences on the surface of the various mold inserts.

Cooling channel geometry	Conventional	Milled	Conformal	Conventional
Mold insert material	1.2311	1.2311	MS1	Ampcoloy 940
Max. surface temperature (°C)	52.6	48.6	47.2	42.5
Average surface temperature (°C)	46.4	44.3	43.2	41.5
Temperature difference on the surface (°C)	9.9	6.6	5.8	1.8

**Table 4:** The thermal properties of the air gap.

Density (kg/m^3^)	1.079
Specific heat (J/kgK)	1008
Thermal conductivity (W/m·°C)	0.02822
Kinematic viscosity (m^2^/s)	17.44 × 10^−6^
Coefficient of thermal expansion (1/°C)	0.003104

## Nomenclature

ρ	density, kg·m^−3^	v	velocity, ms^−1^
t	time, s	p	pressure, Pa
η	dynamic viscosity Pas	g	gravitational acceleration, m·s^−2^
c_p_	specific heat, Jkg^−1^·K^−1^	T	temperature K
∆T	temperature difference, K	λ	thermal conductivity, Wm^−1^·K^−1^
β	polymer expansivity, K^−1^	σ_0_	Stefan-Boltzmann constant, Wm^−2^·K^−4^
ε	emissivity, -	A	cross section, m^2^
δ	thickness, m	α	heat transfer coefficient, Wm^−2^·K^−1^
